# Network pharmacology combined with molecular docking and *in vitro* verification reveals the therapeutic potential of *Delphinium roylei* munz constituents on breast carcinoma

**DOI:** 10.3389/fphar.2023.1135898

**Published:** 2023-09-01

**Authors:** Wajahat Rashid Mir, Basharat Ahmad Bhat, Ashish Kumar, Rohan Dhiman, Mustfa Alkhanani, Abdullah Almilaibary, Mohd Younis Dar, Showkat Ahmad Ganie, Manzoor Ahmad Mir

**Affiliations:** ^1^ Department of Bio-Resources, School of Biological Sciences, University of Kashmir, Srinagar, Jammu and Kashmir, India; ^2^ Department of Life Science, National Institute of Technology, Rourkela, Odisha, India; ^3^ Department of Family and Community Medicine, Faculty of Medicine, Al Baha University, Al Bahah, Saudi Arabia; ^4^ Department of Biology, College of Science, Hafr Al Batin University of Hafr Al-Batin, Hafar Al Batin, Saudi Arabia; ^5^ Regional Research Institute of Unani Medicine (RRIUM), University of Kashmir, Srinagar, Jammu and Kashmir, India; ^6^ Department of Clinical Biochemistry, School of Biological Sciences, University of Kashmir, Srinagar, Jammu and Kashmir, India

**Keywords:** *Delphinium roylei*, breast cancer, HR/LC-MS, 8-hydroxycoumarin, anticancer activity, network pharmacology, molecular docking and MD simulations

## Abstract

*Delphinium roylei* Munz is an indigenous medicinal plant to India where its activity against cancer has not been previously investigated, and its specific interactions of bioactive compounds with vulnerable breast cancer drug targets remain largely unknown. Therefore, in the current study, we aimed to evaluate the anti-breast cancer activity of different extracts of *D. roylei* against breast cancer and deciphering the molecular mechanism by Network Pharmacology combined with Molecular Docking and *in vitro* verification. The experimental plant was extracted with various organic solvents according to their polarity index. Phytocompounds were identified by High resolution-liquid chromatography-mass spectrometry (HR-LC/MS) technique, and SwissADME programme evaluated their physicochemical properties. Next, target(s) associated with the obtained bioactives or breast cancer-related targets were retrieved by public databases, and the Venn diagram selected the overlapping targets. The networks between overlapping targets and bioactive were visualized, constructed, and analyzed by STRING programme and Cytoscape software. Finally, we implemented a molecular docking test (MDT) using AutoDock Vina to explore key target(s) and compound(s). HR-LC/MS detected hundreds of phytocompounds, and few were accepted by Lipinski’s rules after virtual screening and therefore classified as drug-like compounds (DLCs). A total of 464 potential target genes were attained for the nine quantitative phytocompounds and using Gene Cards, OMIM and DisGeNET platforms, 12063 disease targets linked to breast cancer were retrieved. With Kyoto Encyclopaedia of Genes and Genomes (KEGG) pathway enrichment, a total of 20 signalling pathways were manifested, and a hub signalling pathway (PI3K-Akt signalling pathway), a key target (Akt1), and a key compound (8-Hydroxycoumarin) were selected among the 20 signalling pathways via molecular docking studies. The molecular docking investigation revealed that among the nine phytoconstituents, 8-hydroxycoumarin showed the best binding energy (−9.2 kcal/mol) with the Akt1 breast cancer target. 8-hydroxycoumarin followed all the ADME property prediction using SwissADME, and 100 nanoseconds (ns) MD simulations of 8-hydroxycoumarin complexes with Akt1 were found to be stable. Furthermore, *D. roylei* extracts also showed significant antioxidant and anticancer activity through *in vitro* studies. Our findings indicated for the first time that *D. roylei* extracts could be used in the treatment of BC.

## Graphical Abstract

**Figure:**
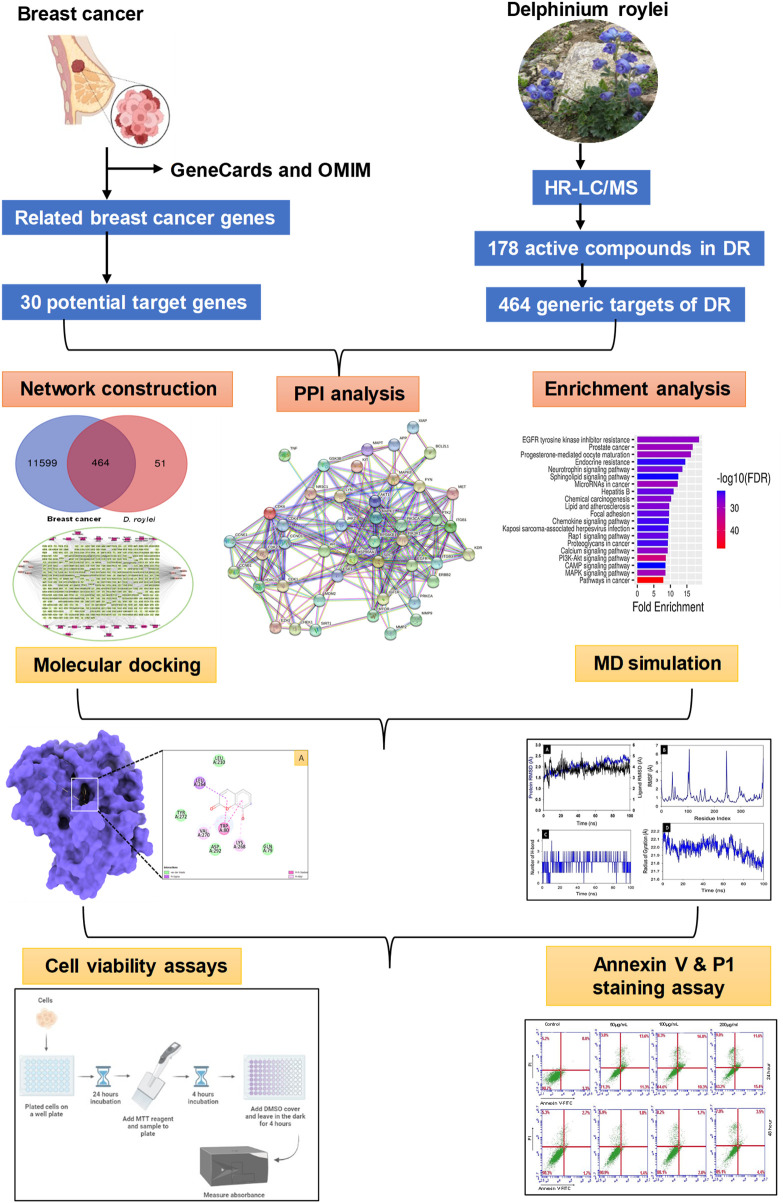
The graphical abstract illustrates a multidisciplinary approach to uncover the therapeutic promise of Delphinium roylei Munz, a medicinal plant, against breast carcinoma.

## Introduction

Breast cancer (BC) is the most commonly diagnosed cancer worldwide, and its burden has been rising over the past decades (Pistelli et al., 2021; Pospelova et al., 2022). Having replaced lung cancer as the most commonly diagnosed cancer globally, breast cancer today accounts for 1 in 8 cancer diagnoses and a total of 2.3 million new cases in both sexes combined (Sung et al., 2021). Representing a quarter of all cancer cases in females, it was by far the most commonly diagnosed cancer in women in 2020, and its burden has been growing in many parts of the world, particularly in transitioning countries (Heer et al., 2020). An estimated 685,000 women died from breast cancer in 2020, corresponding to 16% or 1 in every 6 cancer deaths in women (Deo et al., 2022). The discovery of new cancer treatments is still regarded as an active research area despite the great advance in the area of chemotherapy. Medicinal plants are regarded as a renewable source of bioactive compounds that can be exploited in the treatment of various ailments including cancer (Tariq et al., 2021; Mir et al., 2022a; Mir et al., 2022b). Phytochemicals play an important role in the initiation, development, and advancement of carcinogenesis, as well as in suppressing or reversing the early stages of cancer or the invading potential of premalignant cells and also regulate cell proliferation and apoptosis signalling pathways in transformed cells (George et al., 2021; Bhat et al., 2022a; Bhat et al., 2022b).

*Delphinium roylei* Munz. is an important medicinal herb of the Delphinium genus. The ethnomedicinal uses of this plant include treating the liver and persistent lower back discomfort (Sharma and Singh, 1989; Kumar and Hamal, 2011). Diterpenoid alkaloid and flavanols, which constitute most of the compounds isolated from Delphinium plants, have been tested for various biological activities, such as effects on cholinesterase inhibition, antimicrobial, antineoplastic, insecticidal, anti-inflammatory, and anticancer activities (Yin et al., 2021). Delphinidin is a type of anthocyanin isolated from genus *Delphinium*, which has anticancer, anti-inflammatory, and anti-angiogenic properties. Recent *in-vitro* studies showed that delphinidin can inhibit the invasion of HER-2-positive MDA-MB-453 breast cancer cell line, with low cytotoxicity on normal breast cells (Wu et al., 2021) and ovarian cancer cells (Lim et al., 2017) and can also induce autophagy in breast cancer cells (Chen et al., 2018).

Due to the complex nature of plant extracts and their chemical constituents, it is difficult to understand the molecular mechanism by which they act on certain molecular targets due to the synergistic effects of their chemical constituents and the fact that they could interact with many targets simultaneously. In recent years, network pharmacological analysis has been effectively applied for prediction of the protein targets and the related disease pathways of plant active constituents.

Network pharmacology, based on system biology, includes network database retrieval, virtual computing, and high throughput omics data analysis. This approach breaks the ancient limitation of one drug–one biological target research and is applied mainly to explaining effective mechanisms, active ingredient screening, and pathogenesis research (Yu et al., 2021).

The versatile approach of molecular docking, which is based on the theory of ligand-receptor interactions, is widely used in drug discovery to understand how compounds bind with their molecular targets (Tiwari and Singh, 2022; Saifi et al., 2023).

Here, in the present study, we carried out a HR/LC-MS analysis to identify the phytoconstituents present in the *D. roylei* and screened to find drug-likeness compounds by ADMET analysis. Secondly, we used network pharmacology to predict the potential effective components, corresponding target genes, and pathways of phytocompounds of *D. roylei* against breast cancer. Lastly, we explore the molecular mechanism of the most potent bioactive constituent of *D. roylei* and a hub therapeutic target to alleviate the breast cancer based on molecular docking, molecular dynamic (MD) simulation and *in vitro* experimental analysis.

## Materials and methods

### Collection of plant material

The root parts of the *Delphinium roylei* plant were taken from high-altitude areas of Kashmir Himalaya. The collected samples were identified and confirmed by Akhtar Hussain Malik, Taxonomist, Department of Botany, the University of Kashmir, with voucher specimen No.2954-(KASH).

### Extraction

Various solvents such as methanol, ethanol, ethyl acetate, and petroleum ether were selected as extraction solvents according to their polarity index to obtain plant extract using the Soxhlet equipment technique. About 200 g of the *D. roylei* plant material was powdered using a mechanical grinder after being cleaned with deionized water, dried in the shade for 15 days, pulverized with a mechanical grinder, and placed in an airtight container. The extracts were concentrated using a rotating vacuum evaporator after being filtered using Whatman no. 1 filter paper. They were then kept at 4°C for further use.

### High resolution-liquid chromatography-mass spectrometry (HR/LC-MS)

The HR/LC-MS analysis of the ethanolic extract was carried out by a UHPLC-PDA-Detector Mass Spectrophotometer (HR/LC-MS 1290 Infinity UHPLC System), Agilent Technologies^®^, Santa Clara, CA, USA (Noumi et al., 2020). It consisted of a HiP sampler, binary gradient solvent pump, column compartment, and Quadrupole Time of Flight Mass Spectrometer (MS Q-TOF) with a dual Agilent Jet Stream Electrospray (AJS ES) ion source. A total of 1% formic acid was used as a solvent in deionized water (solvent A) and acetonitrile (solvent B). The flow rate of 0.350 mL/min was used, while MS detection was performed in MS Q-TOF. Compounds were identified via their mass spectra and their unique mass fragmentation patterns. Compound Discoverer 2.1, ChemSpider, and PubChem were the main tools for identifying the phytochemical constituents.

### Network pharmacology-based analysis

Eight 8) compounds were identified according to UPLC-MS/MS analysis and were subjected to network pharmacology-based analysis. The identification of the target genes linked to the selected constituents was performed using the database STITCH DB (http://stitch.embl.de/,ver.5.0) and the obtained results were utilized for construction of compound-target (C-T) network using Cytoscape 3.5.1. Cytoscape combined score of interactions was adopted for judging the importance of nodes in each given network. Information about functional annotation and the pathways that were highly associated with the target proteins were retrieved from DAVID ver. 6.8 (Database for Annotation, Visualization and Integrated Discovery) and the Kyoto Encyclopedia of Genes and Genomes (KEGG) pathways. Relevant pathways with *p*-values <0.05 were selected. Target-pathway and constituent-Pathway networks were constructed to visualize the interactions between compounds, targets and cancer-related pathways.

### In-silico drug-likeness and toxicity predictions

In silico drug-likeness and toxicity of top hit compounds in the database based on were carried out using the SwissADME web browser (http://www.swissdme.com) (Daina et al., 2017). Drug-likeness and toxicity filtering was based on Lipinski’s rule of five (Lipinski, 2004). For example, constituents with predicted oral bioavailability (OB) ≥30 were considered active. Constituents that satisfied less than three criteria were considered inactive.

### Molecular docking

The ligand molecule (8-hydroxycoumarin) are retrieved in the form of 3D Standard Data Format from the PubChem database (3D SDF) (Bolton et al., 2011). The ligand was then converted from 3D SDF to Protein Data Bank (PDB) format using Avogadro software. The crystal structures of breast cancer target proteins (Akt1, SRC, EGFR, IL6, HSP90AA and ESR1) were retrieved from the Protein Data Bank. Biovia Discovery studio was used to eliminate undesirable bindings, ligand molecules, water molecules, and other contaminants from the macromolecule. After that polar hydrogens were added to the protein throughout the preparation process for improved interactions, followed by Kollman charges and other modifications. Molecular docking studies were performed between the target proteins of breast cancer and compounds of the selected plant using AutoDock version 4.2.1 (Trott and Olson, 2010). All other parameters were left at their default values. the grid box was generated centered at X = 14.09, Y = 15.47, Z = 15.48 with dimensions X:3.53, Y:0.58, Z: 12.41. The Lamarckian Genetic Algorithm (LGA) was used for docking studies on the protein and ligand complexes (Fuhrmann et al., 2010). The RMSD clustering maps were developed after the docking process was complete by re-clustering with the clustering tolerances of 1, 0.5, and 0.25 to identify the best cluster with the most populations and lowest energy score.

### Molecular dynamics (MD) simulation study

MD simulations were performed using the Desmond 2020.1 from Schrodinger, LLC (Chow et al., 2008; Release, 2017) on the dock complex of Akt1 and 8-hydroxycoumarin ligand. This system used the OPLS4. force field (Mazurek et al., 2021) and an explicit solvent model containing TIP3P water molecules in a period boundary salvation box of 10 Å × 10 Å x 10 Å dimensions. The system was initially equilibrated to retrain over the protein-ligand complexes using an NVT ensemble for 10 ns. Following the preceding phase, an NPT ensemble was used to carry out the brief run of minimization and equilibration for 12 ns. The NPT ensemble was assembled using the Nose–Hoover chain coupling method and operated at 27°C for 1.0 ps under a pressure of 1 bar for the duration of the investigation. The time step was 2 fs. The Martyna–Tuckerman–Klein barostat method with a 02 ps relaxation time was adopted for pressure regulation. The radius for coulomb interactions was set at 9 nm, and long-range electrostatic interactions were calculated using Ewald’s particle mesh approach. With each trajectory, the bonded forces were estimated using the RESPA integrator for the time step of 2 fs. Root mean square fluctuation (RMSF), root mean square deviation (RMSD), solvent accessible surface area (SAS Area), and radius of gyration (Rg) were estimated to monitor the stability of molecular docking simulations (Rapaport and Rapaport, 2004).

### *In vitro* antioxidant activity

#### 1, 1-diphenyl-2-picrylhydrazyl radical scavenging activity

The capacity of the D. roylei extracts to scavenge the DPPH radicals were assessed by using the Gyamfi et al. method with slight modification (Obi et al., 2022). 0.5 mL of a test extract aliquot at various concentrations of 20–160 μg/mL in methanol was dissolved with 0.5 mL of a 100 mM DPPH solution. The resulting absorbance was measured at 517 nm after a 30-min incubation period in complete darkness and at room temperature. The following formula was used to compute the percentage inhibition:

$$\text{Percentage inhibition}:\left({\text{Absorbance}}_{\text{conrol}}-{\text{Absorbance}}_{\text{sample}}\right)\;x\;100/{\text{absorbance}}_{\text{control}}$$

#### Hydroxyl radical scavenging activity

Hydroxyl radical scavenging activity was determined by Elizabeth and Rao with a bit of modification (Nwakaego et al., 2019). The assay measures the 2-deoxyribose breakdown product by condensing it with Thiobarbituric acid. Hydroxyl radicals are produced by the Fenton reaction, which includes a ferric ions-EDTA-ascorbic acid-H_2_O_2_system. The reaction mixture contains these above components and different plant extract concentrations (10–80 μg/mL). 0.5 mL of the reaction mixture was dissolved in 1 mL of 2.8 percent of TCA after 1-h incubation at 37°C, then 1 mL of 1 percent aqueous TBA was poured, and the mixture was then incubated for 15 min at 90°C to develop the color. The absorbance was calculated at 532 nm. Butylated hydroxytoluene (BHT) was used as standard.

$$Scavenging\;effect:\left({Absorbance}_{conrol}-{Absorbance}_{sample}\right)\;x\;100/{absorbance}_{control}$$

#### Reducing power

The assay was carried out using Oyaizu’s method (Zhang et al., 2021). This method estimated the reduction of Fe_3_^+^-Fe_2_^+^ by measuring the absorbance of Pearl’s Prussian blue complex. This procedure relies on the stoichiometric reduction of (Fe_3_^+^) ferricyanide relative to antioxidants. Various concentrations of the plant extracts (1–200 μg/mL) were added to 2.5 mL of 1% potassium ferricyanide [K_3_Fe (CN)_6_] and 2.5 mL of 0.2 M phosphate buffer with a pH of 6.6. Then 2.5 mL of 10% trichloroacetic acid was added to the mixture after 20 min of incubation at 50°C, and the mixture was then centrifuged at 3,000 rpm for 10 min. The upper layer (2.5 mL) was dissolved in 2.5 mL of distilled water and 0.5 mL of 0.1% FeCl_3_, and the absorbance was determined at 700 nm. Rutin was taken as a positive antioxidant, and the reducing power was calculated according to the absorbance values.

#### Superoxide radical scavenging activity

This assay was based on the extract’s capacity to reduce formazan production by scavenging the radicals produced by the riboflavin-NBT system (Jaganathan et al., 2018). The reaction mixture consists of 20 μg riboflavin, 50 mM phosphate buffer with a pH of 7.6, 0.1 mg/3mL NBT, and 12 mM EDTA. The reaction was initiated by illuminating the above reaction mixture with various concentrations of plant extracts (10–80 μg/mL) for 90 s. Then the absorbance was estimated at 590 nm. BHT was used standard antioxidant. The %age of scavenging of superoxide anion s was calculated using the equation.

$$\text{Percentage Inhibition}:\left(1-A_{S}/A_{C}\right)\;x100$$

Where A_S_ is the absorbance of the test sample, and A_C_ is the absorbance of the control used.

### *In vitro* anticancer activity

#### Cell culture

MCF-7, MDA-MB-231, and MDA-MB-468 cell lines were purchased from the National Centre for Cell Science (NCCS) in Pune. Prof. Annapoorni Rangarajan, IISC, Bangalore, kindly supplied the 4T1 cell line. Cells were grown in high glucose media DMEM using 10% FBS and 1% penicillin/streptomycin. The cells were cultured in a CO2 incubator (5%) at 37°C.

#### Cytotoxicity assay

The MTT (3-(4,5-Dimethylthiazol-2-yl)-2,5-diphenyltetrazolium bromide) assay was utilized to determine the cell cytotoxicity (Rezadoost et al., 2019). Breast cancer cells were seeded in 96 well plates with the cell number of 3 × 10^3^ cells in each well and allowed to adhere overnight. To prepare the stock solution, From the stock solution 10 mg/mL, various concentrations (12.5–400 μg/mL) of various extracts of *Delphinium roylei* were obtained in fresh media. Then the breast cancer cells (MCF-7, MDA-MB-231, MDA-MB-468 and 4T1) were treated with extracts of *Delphinium roylei* for 72 h and the plate system was placed into the incubator. MTT solution was applied to every well after incubation. The plate was kept in the incubator at 37° C for 4 h under dim lighting. The supernatant was removed after 4 h, and 100 μL of DMSO was added to the purple formazan to dilute it. The ELISA plate reader was used to read the plate at 595 nm. The percentage of cell cytotoxicity was determined using an optical density.

$$Percentage\;Cell\;viability=\left(OD\;of\;test\;sample/OD\;of\;control\right)\times 100$$

#### Annexin V/PI apoptosis detection

We used a BD Biosciences Annexin V apoptosis detection kit to examine the mechanism behind the anticancer effect of D. roylei ethyl acetate extract. MDA-MB-231 was treated with acetate extract for 24 and 48 h. As instructed by the manufacturer, adherent and free-floating cells were all collected and stained with the fluorescent dyes FITC-Annexin V and PI. Flow cytometry was performed at the Department of Biotechnology, National Institute of Technology, Rourkela, Odisha, India, on a BD Accuri™ C6 flow cytometer (Mehraj et al., 2022)

## Results

### Quantification of chemical components of HR/LC-MS

**FIGURE 1:**
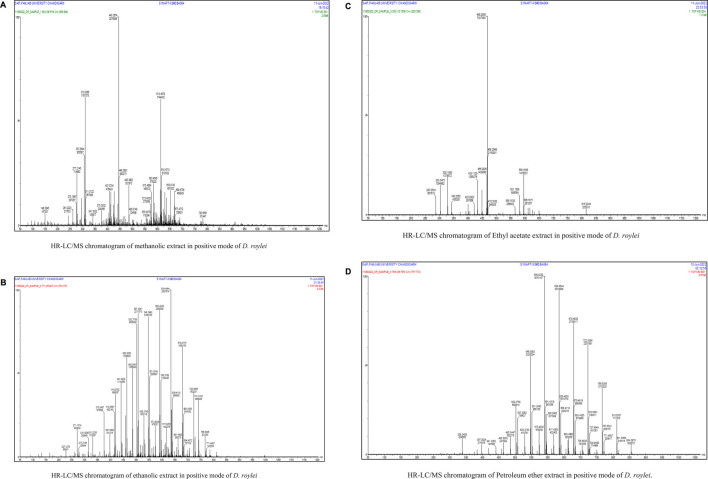
(Continued). **(A)**. HR-LC/MS chromatogram of methanolic extract in positive mode of *D. roylei* **(B)** HR-LC/MS chromatogram of ethanolic extract in positive mode of *D. roylei* **(C)** HR-LC/MS chromatogram of Ethyl acetate extract in positive mode of *D. roylei* **(D)** HR-LC/MS chromatogram of Petroleum ether extract in positive mode of *D. roylei*.

In our previous investigation, 168 phytocompounds were tentatively identified using the chromatography-mass spectrometry (LC/MS) approach in both negative and positive ionization modes (Mir et al., 2022b). A few more compounds were identified by performing the advanced technique like HR-LC/MS) and chromatograms in TOF MS ES + shown in (Figures 1A–D). Figure S1 demonstrates the total ion chromatogram (TIC) of the studied plant’s methanolic, ethanolic, ethyl acetate, and petroleum ether extracts. The examples of a few phytoconstituents identified through HR-LC/MS are 8-hydroxycoumarin, Delsoline, Royleinine, Delsemine B, Herniarin, Aloesin, Talatisamine, Narwedine, Scoparone, and Piperine in *D. roylei* extracts by contrasting their output mass data and retention time relatives to external standards with a reference database and already published data using different software’s. Some identified phytocompounds from chromatograms that possess important pharmacological properties are listed in Table 1. These nine compounds were subjected to network pharmacology, *in silico* docking, and MD simulations analysis. These phytocompounds were identified as chemical markers and listed as potential candidates for further network pharmacology analysis.

**TABLE 1:** List of selected phytocompounds detected in *D. roylei* by HR-LC/MS.

S. No	Compound name	Molecular formula	PubChem ID	Molecular weight
1	8-hydroxycoumarin	C_9_H_6_O3	122783	162.14
2	Delsoline	C_25_H_41_NO_7_	441727	467.6
3	Royleinine	C_24_H_39_NO_5_	101076550	421.6
4	Delsemine B	C_37_H_53_N_3_O_10_	101341025	699.8
5	Herniarin	C_10_H_8_O_3_	10748	176.17
6	Aloesin	C_19_H_22_O_9_	160190	394.4
7	Talatisamine	C_24_H_39_NO_5_	159891	421.6
8	Narwedine	C_17_H_19_NO_3_	10356588	285.34
9	Scoparone	C_11_H_10_O_4_	8,417	206.19
10	Piperine	C_17_H_19_NO_3_	638024	285.34

### Network pharmacology

#### Target gene screening and interaction network construction

**FIGURE 2:**
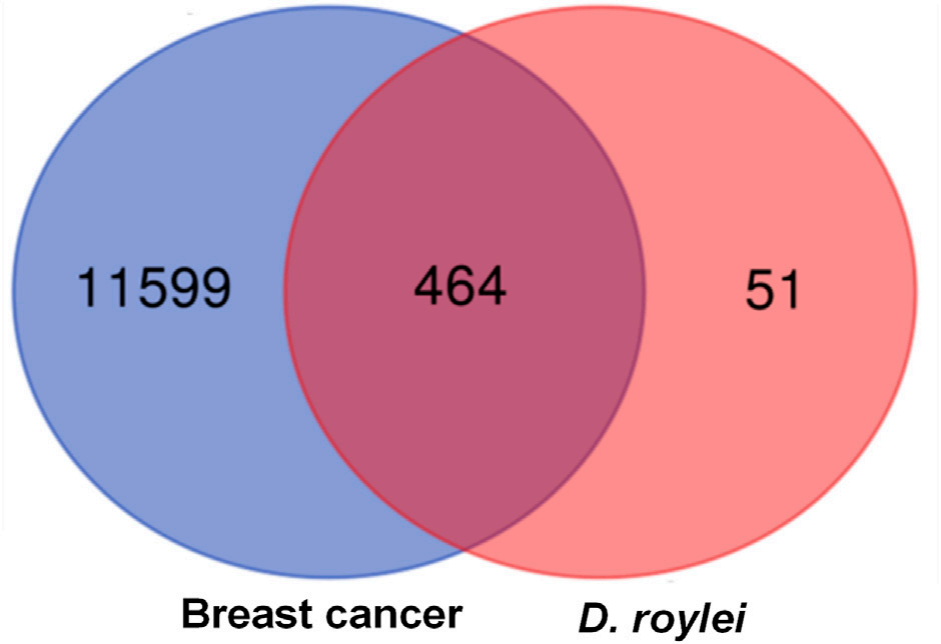
Venn diagram represents common target genes for compound prescription and disease. The size denotes the number of the target genes, the blue circle symbolizes the target genes of breast cancer, the red circle represents the target genes of 10 quantitative components in HR/LC-MS, and the coincident part depicts the common target genes.

**FIGURE 3:**
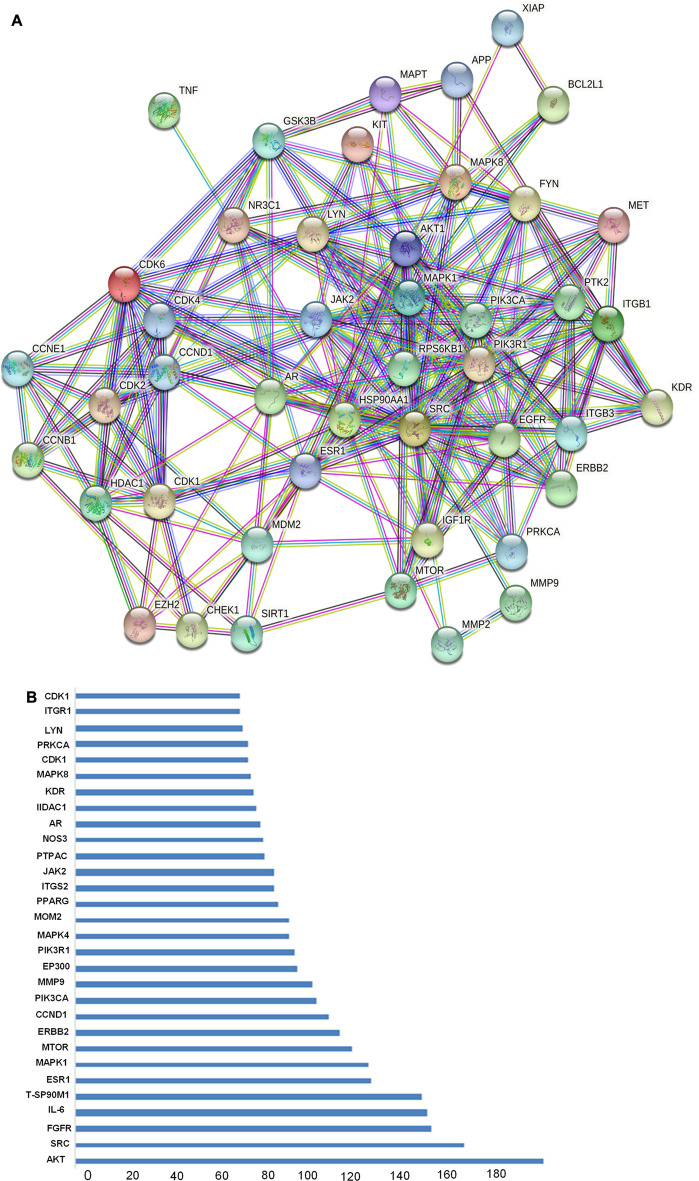
(Continued). The common target genes network interaction results **(A)** PPI network of the common target genes. The nodes represent target genes; the stuffing of the nodes represents the 3D structure of target genes; the edges represent target genes-target genes associations; the colors of the edges represent different interactions; cyan and purple represent known interactions; green, red, and blue-purple represent predicted interactions; chartreuse, black, and light blue represent others.

Total of 514 potential target genes were obtained for the 10 quantitative components of HR/LC-MS (shown in Figure 2). Meanwhile, 12063 disease target genes associated with breast cancer were retrieved using GeneCards, OMIM and DisGeNET platforms. 464 shared common target genes were identified between the quantitative components of HR/LC-MS and breast cancer. All 10 components in *D. roylei*, namely, 8-hydroxycoumarin, Delsoline, Royleinine, Delsemine B, Herniarin, Aloesin, Talatisamine, Narwedine, Scoparone, Piperine in *D. roylei* were targeted for further analysis. The common target genes PPI diagram indicated that there were 464 nodes and 6,035 edges in PPI (Figure 3A). The frequency of occurrence of the top 30 common target genes was shown in Figure 3B. Akt1, SRC, EGFR, IL6, HSP90AA, and other target genes exhibited a high frequency of protein interaction, which may be the node protein of the whole network. The results showed that the selected components of *D. roylei* had a high binding activity with these target proteins and could be used as the potential target genes of HR/LC-MS for treating breast cancer.

#### Screening of key pathways of HR-LC/MS for treating breast cancer

**FIGURE 4:**
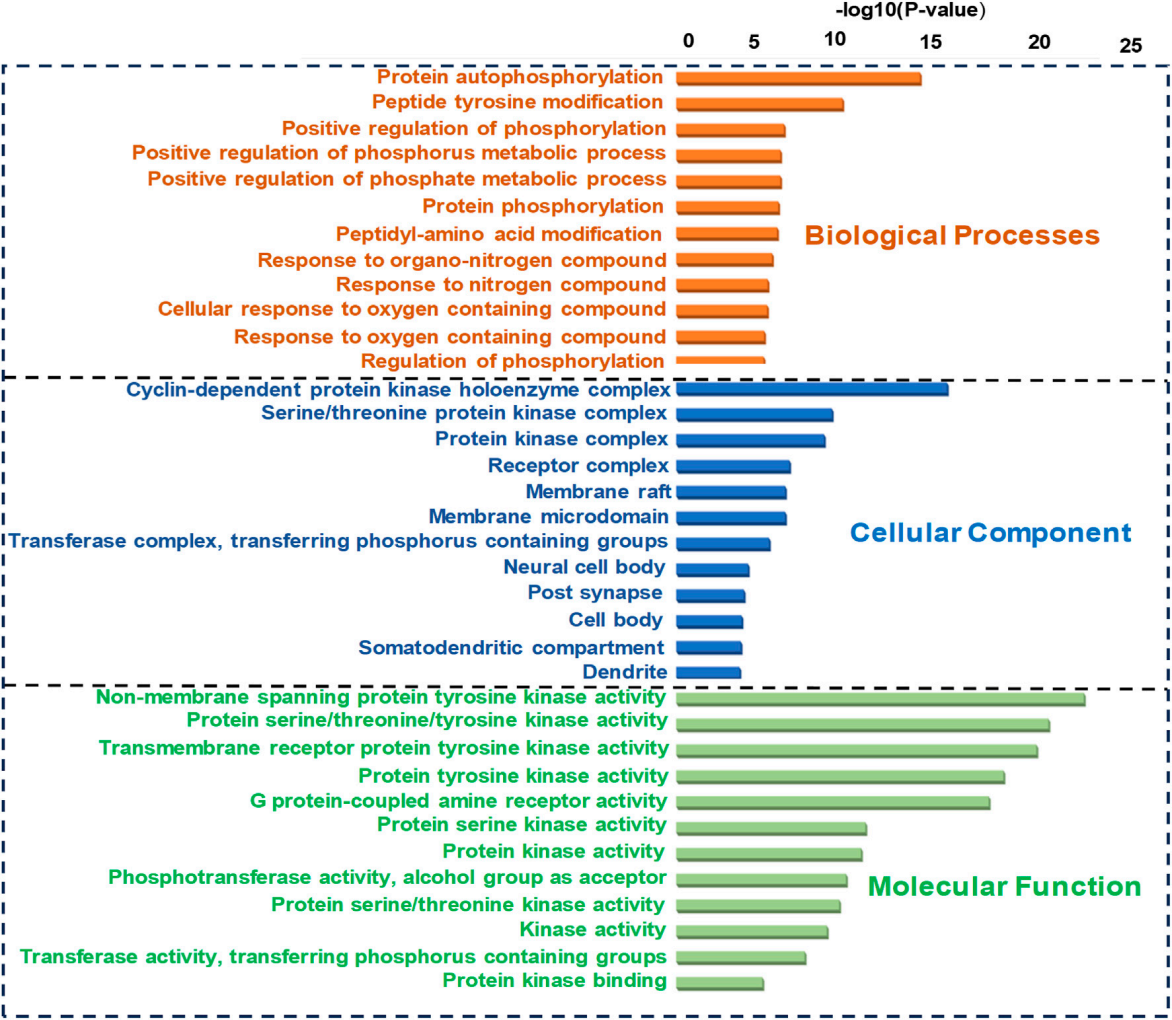
GO (Biological process, molecular function, and cellular component) analysis (top 20). The node length represents the number of target genes enriched, and the node color from blue to red represents the *p*-value from large to small.

**FIGURE 5:**
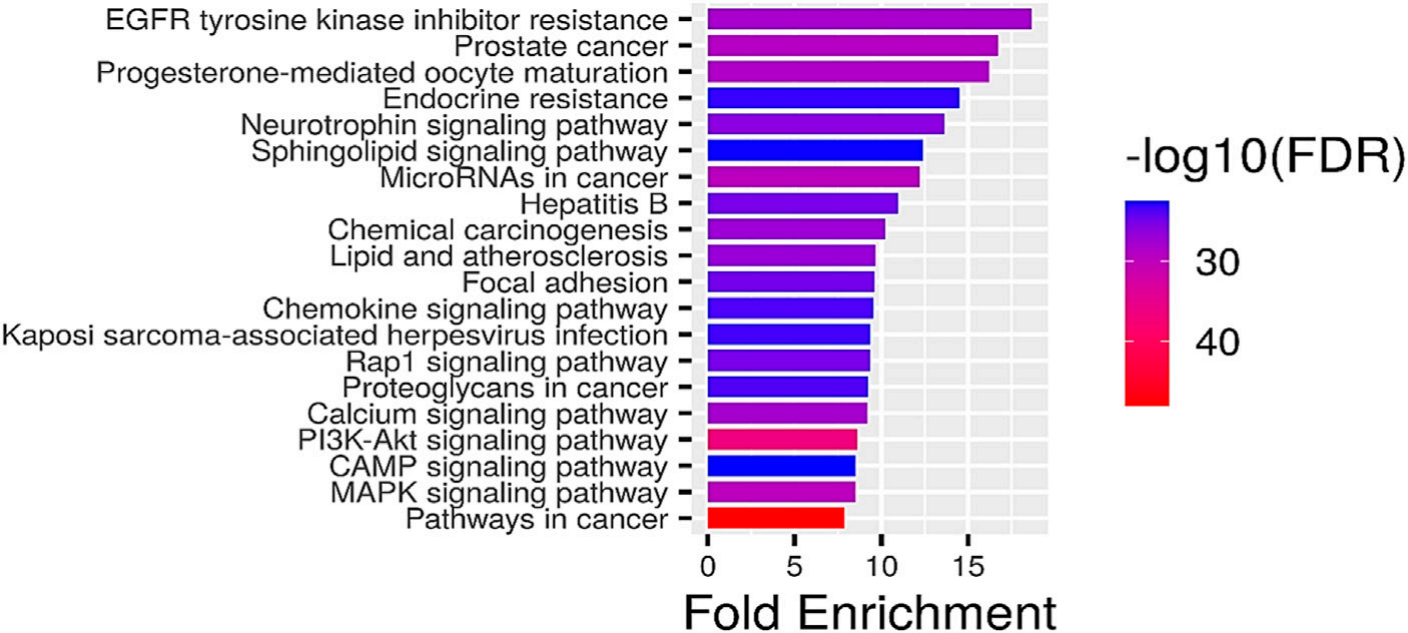
KEGG pathway enrichment analysis (top 20). The node size represents the number of target genes enriched, and the node color from blue to red represents the *p*-value from large to small.

GO analysis of the common target genes showed that the biological process was mainly involved in Protein autophosphorylation, peptidyl-tyrosine modification, and pos. neg. of phosphorylation (Figure 4) and also given as S-2 to S-4. The molecular functions non-membrane spanning protein tyrosine kinase activity and protein serine/threonine/tyrosine kinase activity were leading. The cyclin-dependent protein kinase holoenzyme complex and protein-kinase complex were observed in the cellular component. KEGG pathway enrichment analysis of the aforementioned common target genes is shown in Figure 5 and also given as S-5. After the exclusion of broad pathways, the top 20 signalling pathways are listed in Table 2. This suggested that the effect of HR-LC/MS for treating breast cancer may act on multiple pathways, as well as complex interactions among these pathways.

**TABLE 2:** Pathway enrichment analysis (top 20).

Pathway	nGenes	Enrichment FDR
Pathways in cancer	85	9.57052E-49
PI3K-Akt signallin*g* pathway	62	2.08477E-37
MAPK signaling pathway	51	2.01947E-30
MicroRNAs in cancer	40	3.70614E-30
Prostate cancer	33	7.6318E-30
Progesterone-mediated oocyte maturation	33	2.03033E-29
EGFR tyrosine kinase inhibitor resistance	30	6.62478E-29
Calcium signaling pathway	45	1.22381E-28
Chemical carcinogenesis	41	5.63792E-28
Lipid and atherosclerosis	42	1.26925E-27
Neurotrophin signaling pathway	33	6.99563E-27
Rap1 signaling pathway	40	7.78613E-26
Hepatitis B	36	7.98124E-26
Focal adhesion	39	1.19652E-25
Proteoglycans in cancer	38	2.05389E-24
Chemokine signaling pathway	37	2.8697E-24
Kaposi sarcoma-associated herpesvirus infection	37	4.83818E-24
Endocrine resistance	28	8.41165E-24
Sphingolipid signaling pathway	30	2.65785E-23
CAMP signaling pathway	38	3.29632E-23

#### Compound prescription-active component-disease target gene-pathway interaction network

**FIGURE 6:**
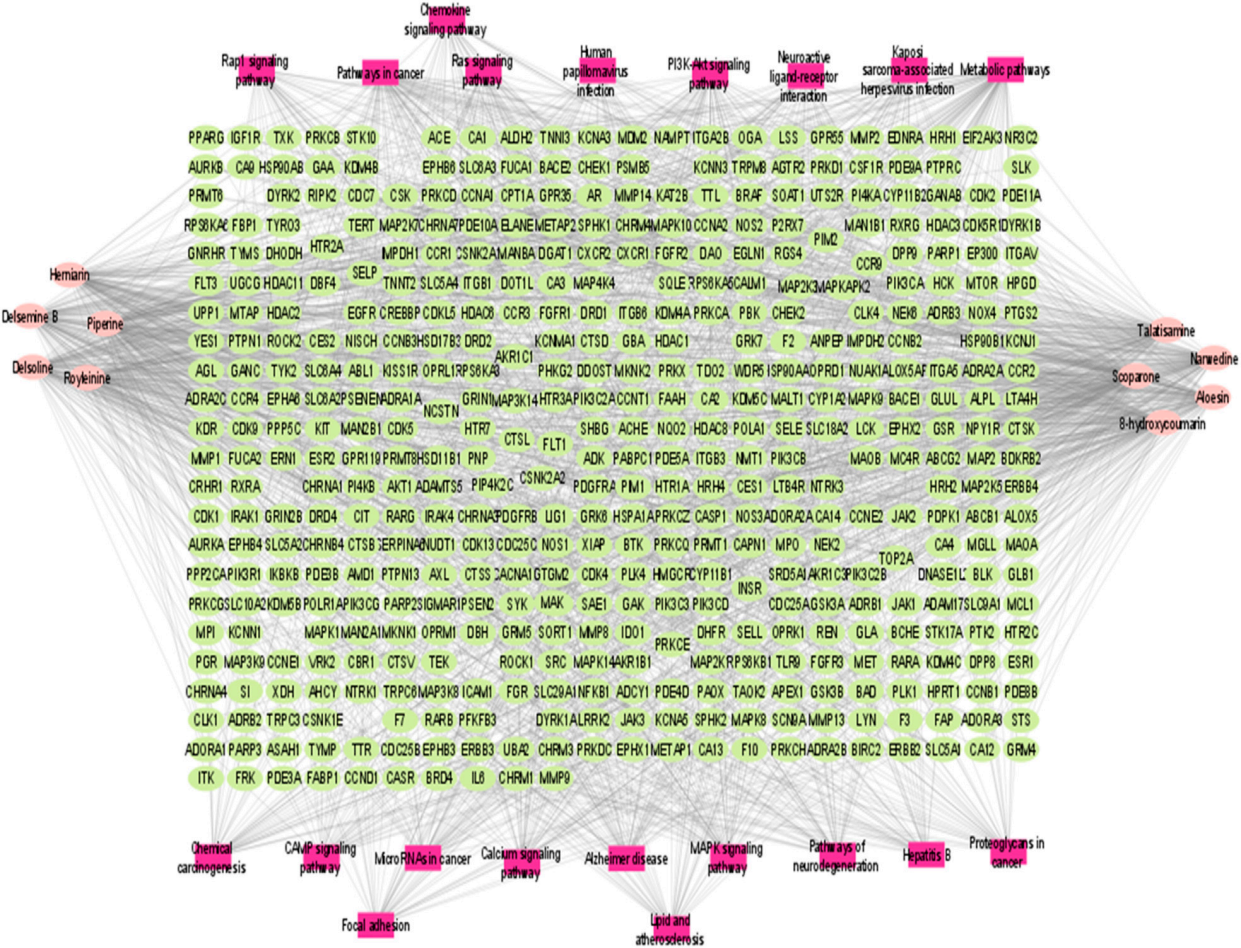
Compound prescription-active component-disease-target gene. The node colors represent different groups: purple represents the components, and blue represents target genes. Component node size represents the degree; low to high degree represents the node size from small to large.

Compound prescription-active component-disease-target gene-pathway interaction network finding is shown in Figure 6. The network contained 474 nodes (464 target genes, 10 active components. Besides, the interaction network results of 10 active compounds are shown in Table 3. The degree of 8-hydroxycoumarin, Delsoline, Royleinine, Delsemine B, Herniarin, Aloesin, Talatisamine, Narwedine, Scoparone, and Piperine were 112, 104, 101, 104, 102, 108, 101, 105, 102 and 110 respectively. The results above show that quality markers in LC-MS may act on the whole biological network system rather than on a single target gene.

**TABLE 3:** Interaction network details of 21 active components.

Component	Degree	Target genes
8-hydroxycoumarin	112	*CCNB3 CDK2 CCNE2 CDK9 CCND1 CDK5R1 DAO CA12 CA9 CA2 MPI EGFR GPR35 AKR1B1 PDGFRB FLT4 IGF1R INSR AURKB PTK2 PLK1 CSNK2A1 CA4 PLK4 TEK AKT1 BACE1 MAP3K8 EPHB4 HSPA1A NUAK1 SQLE FGR LYN METAP2 SRC GSR CYP1A2 ERBB2 GSK3B MAOA ADORA1 PSMB5 IDO1 TDO2 CA1 ESR1 CA14 DBH NFKB1 BRAF CA3 KDR HSD17B3 AURKA MET ACHE CA13 KAT2B XDH PARP1 F2 ADRA2A ADRA2C ADRA2B HTR2A NISCH ADRA1A HTR1A SLC6A3 PDPK1 CDK1 CDK5 ALDH2 GSK3A PRKCG PRKCD PRKCA PRKCB PRKCZ PRKCE PRKCH NQO2 RPS6KA3 MAP2 TYMS GRM4 KCNA3 GRK6 ESR2 CLK1 CCNA1 CCNT1 CDK1 CDK2 CDK4 CDK5 TNNT2 CCNA2 CCNB1 CCNE1 TNNI3 CCNB2 TNNC1 CA6 CA5B CA5A CA7 GPR84 CHRM5 NQO1 HTR2B*
Delsoline	104	*CDK2 CCNE2 GBA AGL UGCG GAA SI GLB1 GANAB FUCA1 FUCA2 GANC GLA KCNJ1 MTAP MAN2B1 CHRM1 EGFR BACE1 CCR1 CA2 PNP PIK3CA MAN2A1 ADRB1 SLC5A2 ADRB2 HRH1 CCR3 SLC5A4 OPRL1 MMP8 PDE5A PDE11A PDE10A CDC25B METAP2 CTSD HTR2A AMD1 FLT3 UTS2R ADRB3 SLC5A1 IKBKB SPHK1 TOP2A SPHK2 CCR9 OGA ANPEP MAPK9 MANBA CHRNA4 SCN9A HSP90AB1 FLT1 KIT KDR CHRM3 HPRT1 LRRK2 ADCY1 CDC25A LSS SRC NOS1 NOS3 CDC25C TTR PRKCQ UPP1 TLR9 RPS6KB1 CACNA1G AURKA ACE ADORA1 ADORA3 HDAC1 HRH4 TTL CASR DRD1 DPP8 ALOX5 MMP13 MMP1 MAP2K7 NUDT1 IRAK4 CCNA1 CDK2 CCNA2 CCNE1 GBA2 MGAM SLC47A2 HTR4 YARS CHRM2 TRPV3 HTR2B DPP7*
Royleinine	101	*GRIN1 CHRM1 GBA CHRM3 AGL KCNJ1 BACE1 PNP PDE5A PDE11A PDE10A FUCA1 CHRNA4 UGCG GAA LRRK2 HTR1A ADRB2 ADRB1 ADRB3 KCNA5 GANAB SLC18A2 CDC25B OPRL1 MTAP MAN1B1 MAN2A1 PABPC1 NOS1 NOS3 BRD4 SLC5A4 CTSD SLC5A1 CREBBP DRD1 NMT1 SLC6A4 UTS2R SI SPHK1 SLC6A2 NR3C2 PGR CCR1 SPHK2 SLC5A2 CA2 DRD4 TTL CCR3 TOP2A CCR2 DPP8 CSF1R XIAP P2RX7 BIRC2 EIF2AK3 ALOX5 NUDT1 CHRM4 PARP1 ERBB2 MAPK8 MAPK9 CACNA1G FAP HTR3A SERPINA6 TERT ROCK2 SHBG IL6 JAK1 ADAM17 PIK3CA GLUL FABP1 CASR GSK3B CDK2 PRMT6 PRMT8 IDO1 PRMT1 SRC GSR GRIN2B CHRM2 GBA2 SLC47A2 HTR4 DPP7 TRPV3 DUSP3 HTR1D MGAM HTR1F GPBAR1*
Delsemine B	104	*CCNE2 F3 PIK3CA CHRNA7 MTOR SLC10A2 IMPDH1 IMPDH2 PDE10A PIK3CD PIK3CB PIK3CG PIK3CA CA2 PDE8B SLC6A3 PIM1 PIM2 EGFR POLA1 MAP3K14 MMP2 CSF1R GNRHR CCR1 ADORA2A DHFR PIK3C2A PIK3C3 SYK MAP3K9 FGR PIK3C2B CCR4 ERBB2 SLC5A2 FGFR3 FGFR1 FGFR2 NAMPT CDK4 SLC5A1 SLC9A1 ADK ABL1 FLT3 BLK DYRK1A PHKG2 LCK MAPK14 SRC MMP13 ERBB4 MMP9 HCK IRAK1 MET MMP14 STK10 MAPK9 SLK MKNK2 FRK GAK TXK DYRK2 STK17A EPHA6 MMP8 MKNK1 AXL RPS6KA6 CSNK1E MAP2K5 RIPK2 DYRK1B CIT EPHB6 ERBB3 MAP2K1 AKT1 TYRO3 SELP MAP4K4 F10 PRKDC DDOST ALOX5AP PPP2CA DOT1L F7 KIT YES1 CDK2 F7 PIK3R1 CCNE1 PIM3 PDE6A HIPK4 SBK1 PHKG1 CSNK1D*
Herniarin	102	*CDK2 CA1 CA12 CA14 CA9 CA4 CA13 EGFR MAOA CA2 AKR1C3 AKR1C1 CA3 KCNA3 SRD5A1 XDH MAOB CBR1 ACHE PARP1 CYP1A2 MET GSK3B AURKA APEX1 GPR35 PTK2 PLK4 MAP3K8 HSPA1A SQLE FGR LYN ALOX5 AURKB PARP2 IGF1R AHCY EP300 KAT2B NOS1 NOS2 NOS3 SRC KDR ADORA1 ADORA2A ESR2 MCL1 ADAMTS5 PTPRC NQO2 MAPK10 RGS4 JAK1 JAK2 TYK2 CDC7 NUDT1 MAP3K14 AKR1B1 KDM4C PDGFRB CES1 MAPK8 CTSK CES2 DYRK2 DYRK1B ADORA3 HSD11B1 GRM4 CAPN1 HMGCR BACE1 HRH2 MGLL MKNK1 SYK TGM2 GRM5 CLK4 RPS6KA5 RPS6KB1 PIM2 PBK BTK NTRK3 PRKX MAP4K4 PDE5A CCNA1 CCNA2 CA7 CA6 CA5A CA5B MCHR1 ADRA1D MIF ADORA2B PIM3*
Aloesin	108	*CDK9 CCNA2 CCND1 ITGB1 ITGAV ITGA2B ITGAV SAE1 AKR1B1 SLC29A1 CA12 MMP9 MMP2 ESR1 ESR2 HDAC6 IGF1R AURKB SRC PTK2 KDR PLK1 HDAC8 HDAC1 MET NEK2 AKT1 NEK6 NUAK1 NOS2 MMP13 CA2 CA4 EGLN1 BACE1 NQO2 HSP90AB1 SORT1 NOX4 DNASE1L3 IKBKB PTPN1 HDAC3 HDAC2 ADORA2A ABCB1 DHODH LIG1 CXCR2 CALM1 HSP90AA1 FBP1 KCNMA1 ERN1 CA1 CA9 HSP90B1 PRKCG PRKCB PRKCZ CDC7 ADRA2C ALOX5 CXCR1 SLC5A1 CBR1 SLC5A2 MCL1 LTB4R SELL SELP PPARG STS SIGMAR1 DRD2 BAD OPRM1 OPRK1 ABCG2 LRRK2 GRK7 TAOK2 MAK CDKL5 VRK2 PIP4K2C CDK13 CSF1R ABL1 CCNT1 CDK2 CDK4 ITGA5 ITGB3 ITGB6 UBA2 CA7 HDAC10 DUSP3 PTGES CA6 VEGFA PSMG3 BCL2 HTR2B HIPK4 ICK*
Talatisamine	101	*GRIN1 CHRM1 PABPC1 CHRM3 AGL GBA BACE1 KCNJ1 SIGMAR1 PNP PDE5A PDE11A PDE10A UGCG GAA MTAP UTS2R CTSD KCNA5 MAN1B1 MAN2A1 LRRK2 CA2 FUCA1 CCR3 ADRB1 CCR1 SPHK2 XIAP BIRC2 PIK3CA P2RX7 DRD1 REN ADRB2 SRC ADRB3 CDC25B HTR7 TOP2A BRD4 FLT3 PDE9A CREBBP TERT NOS1 CACNA1G NOS3 SI NMT1 PRKCB NPY1R ERBB2 PRKCQ MANBA SLC5A4 DRD4 PRKDC PRMT6 CSF1R PRMT8 MKNK2 MKNK1 PRMT1 HTR3A PIM1 PIM2 GANAB PARP1 TTL MAPK8 CHEK2 CDK2 MAPK9 CHEK1 SLC5A2 HSP90AB1 MMP8 CASR NR3C2 PGR ABL1 KIT YES1 ALOX5 LCK MAPK14 CSK GRIN2B CHRM2 HTR4 GBA2 SLC47A2 DPP7 DUSP3 HTR1D PDE1C TRPV3 MGAM HTR1F MAPK11*
Narwedine	105	*CDK1 DBF4 CHRNA3 ROCK2 ACHE OPRM1 OPRD1 BCHE OPRK1 BDKRB2 SLC6A2 SLC6A4 SLC6A3 MAOB DRD4 PAOX MC4R CHRM4 CHRM3 NOS2 CHRNA4 KDM5C MPO KDM4B KDM5B PARP2 JAK3 CHEK2 JAK1 JAK2 ROCK2 PDE10A CHRNA1 PIM1 PIM2 CHEK1 KDM4A ROCK1 POLR1A HTR3A HTR2C HTR1A MAPK8 PRKCD PRKCE PRKD1 RPS6KA5 PRKX PRMT6 IMPDH2 PRMT8 PRMT1 CSNK2A1 CSNK2A2 PRKCQ HTR7 AURKB RPS6KB1 AURKA ADORA1 MALT1 LTA4H DPP8 DPP9 KISS1R WDR5 MAOA CDC25C SYK SIGMAR1 MAPK1 HRH2 PNP KCNA5 NOS1 FAP CDC7 PBK CSF1R PRKCA ESR2 MAP2K1 ADRA2B HRH4 PARP3 EGFR TERT KCNJ1 HRH1 GSK3B MMP13 MMP9 CCNB1 CDC7 CHRNB4 ROCK1 CHRM5 DPP7 ADRA1D PIM3 HTR1F HTR2B HTR1D CHRNB2*
Scoparone	102	*CDK2 CA12 CA9 CA13 CA14 CA1 CA4 XDH CA2 EGFR ALOX5 SRD5A1 CA3 CBR1 SRC MAOA AKR1B1 AKR1C1 GSK3B MAOB ESR2 KCNA3 PTGS2 IGF1R KDR AURKA ERBB2 PDE5A ADAMTS5 PDE3A PDE3B BACE1 GPR35 SQLE FGR LYN PARP1 PARP2 MAPK8 MPO AURKB TYMP GRM4 CDC7 KCNN1 KCNN3 CTSK CTSS CTSL PDGFRB FLT4 INSR TEK EPHB4 MAP2K3 BTK IKBKB MAPK10 CES1 ROCK2 CES2 EPHB3 SYK CLK4 RPS6KA5 RPS6KB1 PIM2 PBK NTRK3 PRKX MAP4K4 PLK1 JAK2 PTPRC TYK2 BRAF HSP90AA1 DYRK1B NQO2 KDM4C MET MAPK14 TGM2 PTPN13 METAP1 ICAM1 SELE CASP1 MAP3K14 APEX1 MCL1 NUAK1 MAPKAPK2 JAK1 CCNA1 CCNA2 CA7 CA6 CA5B CA5A KCNN2 PIM3*
Piperine	110	*PSEN2 CDK2 DBF4 CCNA2 CCNE1 PDGFRA ROCK2 MAOB SIGMAR1 SOAT1 ITK KDM5C KDM4A KDM4C EPHX2 PRKCQ FLT3 SRC JAK2 ADORA2A CSF1R CPT1A FAAH NTRK1 ASAH1 IMPDH2 PDE4D PARP1 CDC7 TRPM8 IRAK4 DGAT1 AURKB ADAMTS5 CDK1 AURKA EDNRA ALPL ACHE ABL1 PFKFB3 STS PDE10A LCK PI4KA SLC5A1 TRPC6 TRPC3 HDAC11 DRD2 NAMPT ADORA3 IDO1 MDM2 GPR119 PPP5C AR EPHX1 PGR ROCK2 GPR55 PDE5A CTSV CTSL RPS6KA3 F10 ADORA1 RPS6KB1 TOP2A ELANE GRM5 CTSB LRRK2 CYP11B1 RARG RARB RARA PDE3A CYP11B2 PDE3B PI4KB CDK5 RXRA RXRG HPGD AGTR2 CRHR1 IKBKB BACE2 CCNA1 CDC7 CDK2 PDGFRB PSENEN ROCK1 CCNA2 NCSTN KDM4E ACACB KDM4D QPCT NAAA SLC6A9 PDE7A HDAC10 SCN2A SCN10A ADORA2B APH1A*

#### Molecular docking studies

**FIGURE 7:**
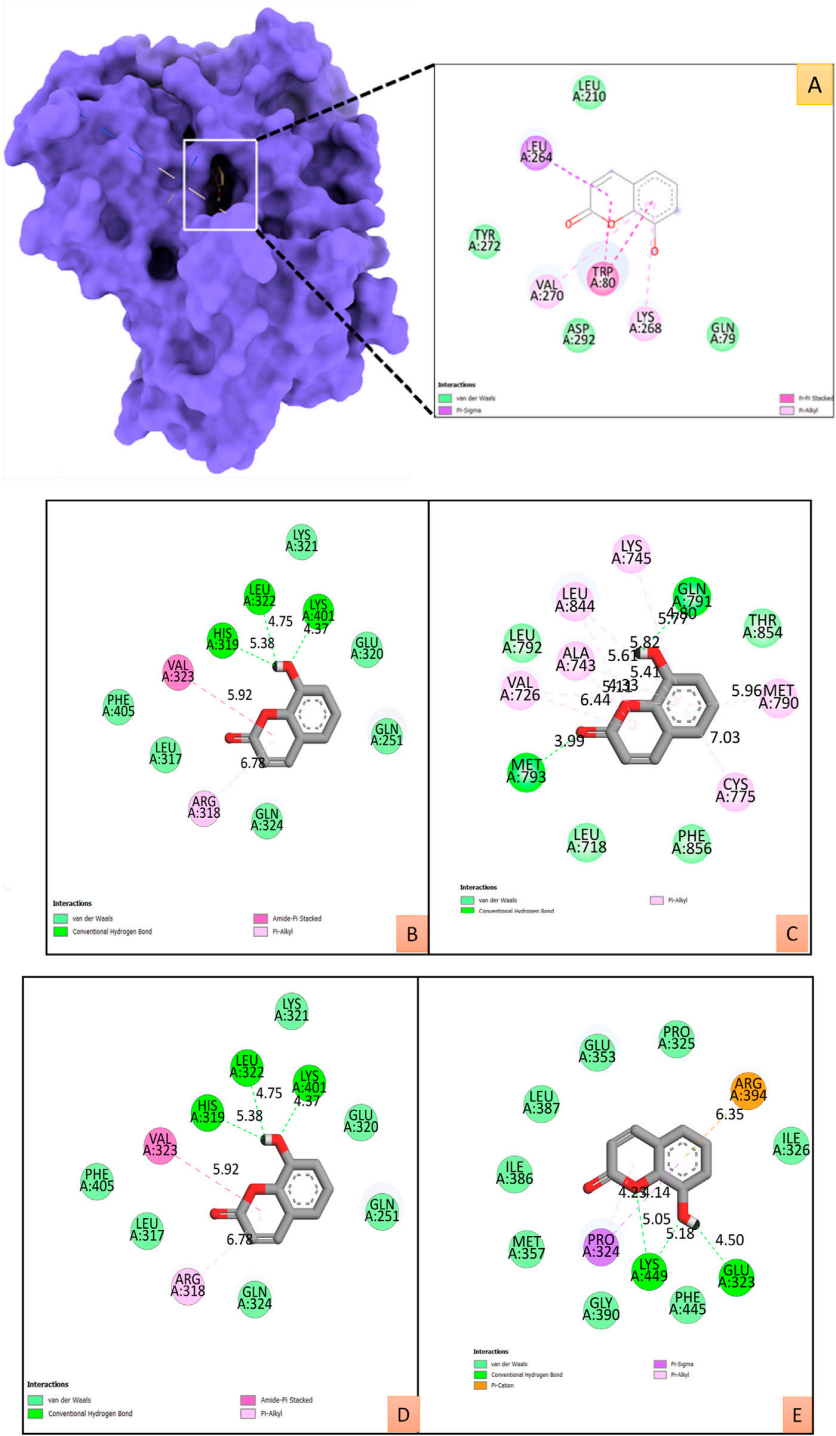
**(A)**. The surface view of the best pose of the Akt1 (PDB ID:4EKL) +8-Hydroxycoumarin complex displays the surface view on the left panel and the 2D interaction profile of the ligand with binding cavity residues. **B-E:** 2D and 3D interactions of 8-Hydroxycoumarin with various target proteins **(B).** SRC (PDB ID:2H8H), **(C).** EGFR (PDB ID: 6DUK), **(D).** IL-6 (PDB ID:1P9M)**, (E).** ESR-1 (PDB ID: 1GWQ) with respective amino acids respectively.

All the binding energy scores are calculated from the best cluster (95%) that falls within the lowest RMSD 0.25 Å. With the lowest binding energy (ΔG–9.2 kcal/mol) and inhibitory concentration, Ki (1.14 µM), 8-hydroxycoumarin showed a considerable binding affinity for the Akt1 (Figure 7A). During the interaction of the ligand 8-hydroxycoumarin, Trp80 is involved in pi-pi stacking, and Lys268 and Val270 residues are involved in pi-alkyl interaction at the binding cavity of the protein. Besides hydrogen bonding, Leu264 is involved in pi-sigma interaction, and the rest residues are van der Waal’s interactions by amino acid residues formed weak non-bonded interaction with the ligand (Figure 7A and right panel). Molecular docking studies were performed to verify the affinity of target protein(s) and bioactive phytocompounds. The 3D interactions of 8-Hydroxycoumarin with various target proteins; SRC, EGFR, IL-6, Hsp90aa1 and ESR-1 and 2D structure of 8-Hydroxycoumarin interacted with respective amino acids respectively are shown in Figures 7B–F. Table 4 depicts the docking and scores of the *D. roylei* compounds; 8-hydroxycoumarin, Delsoline, Royleinine, Delsemine B, Herniarin, Aloesin, Talatisamine, Narwedine, Scoparone, and Piperine against the active sites of the identified protein targets, Akt1, SRC, EGFR, IL-6, Hsp90aa1and ESR-1 performed using the AutoDock Vina software.

**TABLE 4:** Binding energy obtained from the docking calculations of bioactive phytoconstituents with target proteins.

S. No.	Compound name	Akt1 Energy score (kcal/mol)	SRC Energy score (kcal/mol)	EGFR Energy score (kcal/mol)	IL-6 Energy score (kcal/mol)	Hsp90aa1 Energy score (kcal/mol)	ESR-1 Energy score (kcal/mol)
1	8-hydroxycoumarin	−9.2	−5.30	−5.74	−4.06	−5.42	−5.75
2	Delsoline	−6.8	−7.4	−6.3	−5.9	−5.8	−7.2
3	Royleinine	−7.5	−8.1	−7.4	−6.1	−6.3	−6.7
4	Delsemine B	−9	−8.7	−8.2	−7.3	−7.2	−8.2
5	Herniarin	−7.4	−6.6	−5.9	−5.9	−5.5	−6.6
6	Aloesin	−8.1	−7.7	−8.9	−6.9	−6.8	−7.4
7	Talatisamine	−7.5	−7.3	−6.6	−6	−6.2	−7
8	Narwedine	−8.5	−7.1	−7.6	−7	−6.5	−8.6
9	Scoparone	−7.5	−6.5	−6.1	−5.9	−5.3	−6.8
10	Piperine	−9	−8.4	−7.6	−6.7	−7.4	−8

#### Drug-likeness prediction of *D. roylei* phytoconstituents

The compounds retrieved from PubChem were assessed for Lipinski’s Rule of 5, with drug-likeliness properties. Furthermore, ADMET evaluation was applied and was selected for molecular docking to determine the binding affinity with protein at the active site. Almost all the nine phytoconstituents accept Lipinski’s rule with few limitations, as shown in Table 5.

**TABLE 5:** Drug-likeness prediction of *D. roylei* phytoconstituents by ADMET evaluation using SwissADME Software.

Compound Name	MW	HBA	HBD	RB	TPSA (Å^2^)	Lipinski’s Rule	GI absorption	BBB	Solubility	ADMET Screening
8-hydroxycoumarin	162.14	3	1	0	50.44	Yes	High	Yes	soluble	Yes
Delsoline	467.6	8	3	6	100.85	Yes	High	No	Very soluble	Yes
Royleinine	421.6	6	2	4	71.39	No	High	No	soluble	Yes
Delsemine B	699.8	11	4	14	179.39	No	Low	No	soluble	No
Herniarin	176.17	3	0	1	39.44	No	High	Yes	soluble	Yes
Aloesin	394.4	9	5	4	157.66	Yes	Low	No	soluble	Yes
Talatisamine	421.6	6	2	5	71.39	No	High	No	soluble	Yes
Narwedine	285.34	4	0	1	38.77	Yes	High	Yes	soluble	Yes
Scoparone	206.19	4	0	2	48.67	Yes	High	Yes	soluble	Yes
Piperine	284.34	3	0	4	38.77	Yes	High	Yes	soluble	Yes

MW: molecular weight, HBA: hydrogen bond acceptors, HBD: hydrogen bond donors, RB: rotatory bonds, GI: gastrointestinal absorption, BBB: blood brain barrier.

#### Molecular dynamic (MD) simulation study

**FIGURE 8:**
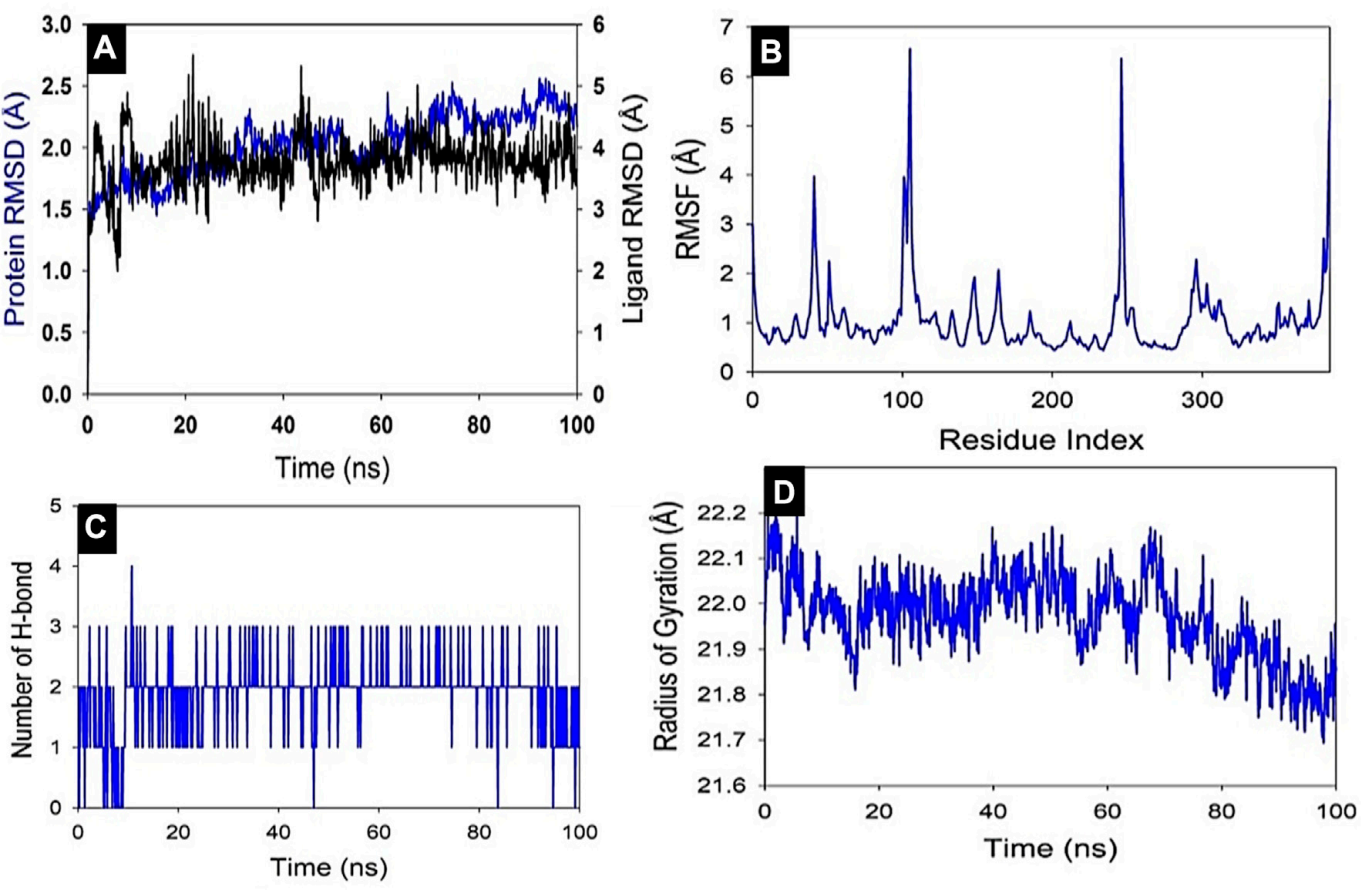
MD simulation analysis of 100 ns trajectories of **(A)** Cα backbone of Akt1 bound to 8-Hydroxycoumarin ligand, **(B)** RMSF of Cα backbone of Akt1 bound to 8-Hydroxycoumarin-ligand **(C)** Radius of gyration (Rg) of Cα backbone of Akt1 bound to 8-Hydroxycoumarin **(D)** Formation of hydrogen bonds in Akt1 bound to 8-Hydroxycoumarin.

Studies using molecular dynamics and simulation (MD) were conducted to determine the convergence and stability of the Akt1+8-Hydroxycoumarin complex. Comparing the root mean square deviation (RMSD) values, the simulation of 100 ns showed stable conformation. The RMSD of the Cα-backbone of Akt1 bound to 8-Hydroxycoumarin showed a deviation of 2.1 Å (Figure 8A), while the ligand showed an RMSD deviation of 3.8.0 Å. Stable RMSD plots indicate good convergence and stable conformations throughout the simulation. As a result, it may be inferred that 8-Hydroxycoumarin bound with Akt1 is quite stable in complex due to the ligand’s increased affinity. The plot for root mean square fluctuations (RMSF) indicates the residual fluctuations due to conformational variations in different secondary structures. Here RMSF plot displayed fluctuating residues while high fluctuations were observed among 50–60, 110–130, and 240–260 residual positions (Figure 8B). The highest fluctuating peaks comprised of 3.8 Å to 6.7–6.8 Å, might be due to higher ordered flexibility conforming into loops (Figure 2B). Therefore, the protein Akt1 has significant flexibility to conform to specific secondary structures to accommodate the ligand. The radius of gyration (Rg) quantifies how compact a protein is with a ligand molecule (Miu et al., 2008). In this investigation, the Akt1 Cα -backbone linked to 8-hydroxycourmarin demonstrated a stable radius of gyration (Rg) values between 22.1 and 21.8 Å, hence there are no sudden changes in radius of gyration as shown in (Figure 8D). This steady values of Rg suggest that despite the structural changes caused by the compounds, the protein remains folded. Significant stable gyration (Rg) suggests that the protein is oriented in a highly compact manner when it is attached to a ligand. The quantity of hydrogen bonds between the ligand and protein indicates the complex’s interaction’s complex stability and depth. During the 100 ns of the simulation, there were significant numbers of hydrogen bonds between Akt1 and 8-hydroxycourmarin (Figure 8C). Between Akt1 and 8-hydroxycourmarin-ligand, the average number of hydrogen bonds is two. The overall analysis of Rg indicates that the binding of the various ligands causes the corresponding proteins to become less flexible and more compact.

#### Molecular mechanics generalized born surface area (MM-GBSA) calculations

Utilizing the MD simulation trajectory, the binding free energy and other contributing energy in the form of MM-GBSA is determined for Akt1 bound to 8-Hydroxycoumarin complexes. The results (Table 6) suggested that the maximum contribution to ΔGbind in the stability of the simulated complexes was due to ΔG_bind_Coulomb, ΔG_bind_vdW, ΔG_bind_Hbond, and ΔG_bind_Lipo, while ΔG_bind_Covalent and ΔG_bind_SolvGB contributed to the instability of the corresponding complex. Akt1 bound to 8-Hydroxycoumarin complex has significantly higher binding free energies dGbind = −52.23 ± 5.12 kcal/mol (Table 6). These results supported the potential of AKT1 bound to 8-Hydroxycoumarin having a high affinity of binding to the protein, efficiency in binding to the selected protein, and the ability to form stable protein-ligand complexes (Balasubramaniam et al., 2021).

**TABLE 6:** Binding free energy components for the Akt1+8-hydoxycoumarin calculated by MM-GBSA.

Energies (kcal/mol)	AKT1 + 8-hydroxycoumarin
ΔG_bind_	−52.23 ± 5.12
ΔG_bind_Lipo	−12.23 ± 2.19
ΔG_bind_vdW	−41.12 ± 1.08
ΔG_bind_Coulomb	−27.41 ± 2.29
ΔG_bind_H_bond_	−3.13 ± 1.32
ΔG_bind_SolvGB	35.41 ± 1.57
ΔG_bind_Covalent	5.91 ± 2.61

### *In vitro* antioxidant activity

#### DPPH assay

**FIGURE 9:**
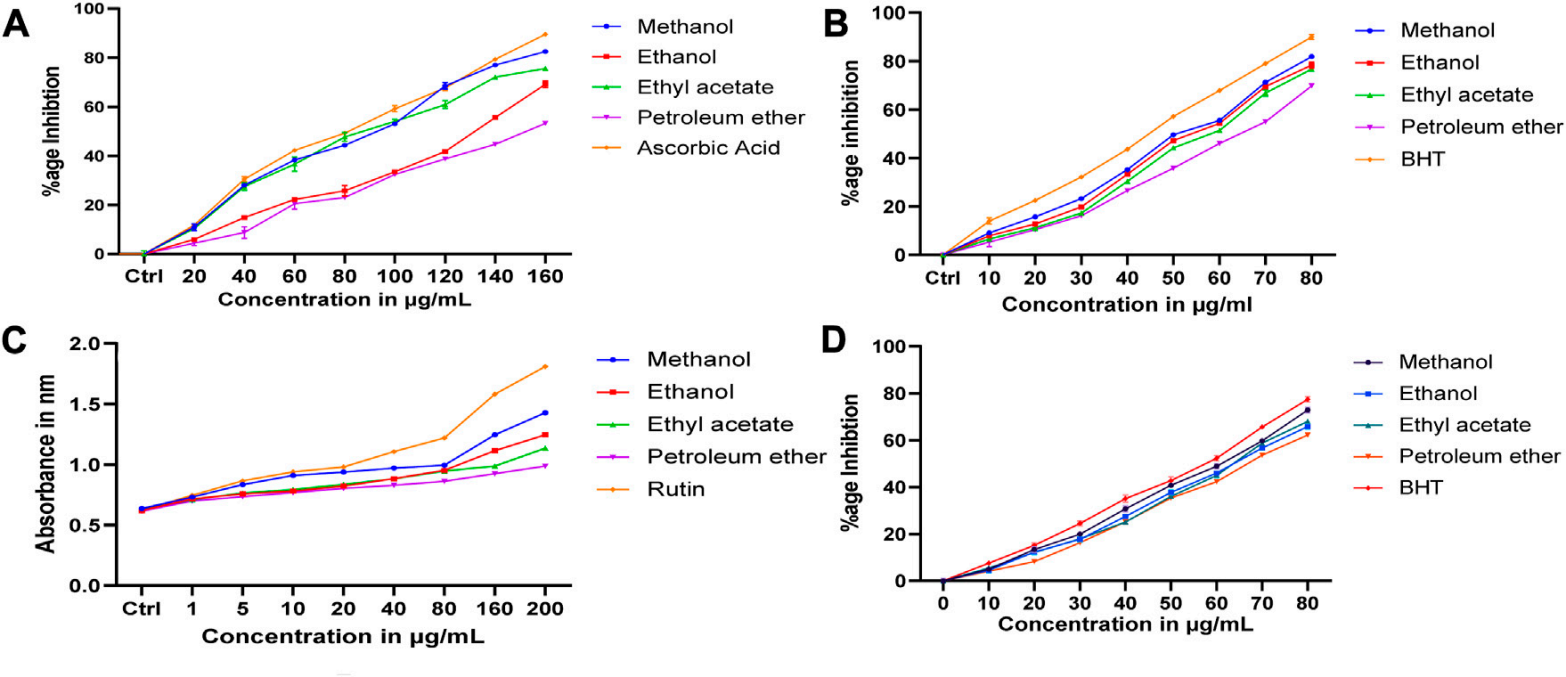
Antioxidant results of *D. roylei* **(A)**. DPPH radical scavenging assay **(B)**. Hydroxyl radical scavenging assay **(C)**. Reducing power assay, and **(D)**. Superoxide radical scavenging assay.

All the extracts showed different levels of DPPH radical scavenging activity over the range of 20–160 μg/mL concentration, as shown in Figure 9A. The methanolic extract exhibited the strongest DPPH radical scavenging activity compared to other extracts. The extract’s radical scavenging activity was effective in the order Methanolic > ethanol > Ethyl acetate > Petroleum ether. A maximum of 82.46% ± 0.2% radical scavenging potential was observed at 200 μg/mL of methanolic extract used, whereas for ascorbic acid, the scavenging activity was 89.57% ± 0.25%. A minimum of 66.35% ± 0.32% scavenging potential was observed at 200 μg/mL of petroleum ether extract. Standards and all the extracts showed a dose-dependent inhibition of the DPPH radicals.

#### Hydroxyl radical scavenging assay

In this assay, results showed that the methanolic extract of *D. roylei* had the highest potential to scavenge hydroxyl radicals than the ethanolic extracts, ethyl acetate, and petroleum ether, as shown in Figure 9B. At a concentration of 80 μg/mL, the methanolic, ethyl acetate, ethanolic, and petroleum ether extract showed the maximum scavenging effect of 81.92% ± 0.49%, 78.72% ± 0.8%, 73% ± 0.7% and 69.88% ± 0.55% inhibition on hydroxyl radicals. Butylated hydroxytoluene (BHT) taken as a control had shown a more scavenging effect (90.07% ± 1%) than plant extracts.

#### Reducing power assay

As illustrated in Figure 9C, Fe_3_^+^ was transformed to Fe_2_^+^ in the presence of *D. roylei* extract and the reference compound Rutin to measure the reductive capability. The reducing power increased with an increase in the concentration of plant extracts. The methanolic extract of *D. roylei* showed significant reducing power when compared with standard Rutin. The reducing power demonstrated by the methanolic extract of the plant was 1.429 ± 0.005 at the concentration of 200 μg/ml as compared to 1.811 ± 0.0035 shown by standard Rutin at the same concentration. Ethanolic, ethyl acetate, and petroleum ether extracts showed less reducing power (1.246 ± 0.0025, 1.136 ± 003, and 0.987 ± 0.005, respectively) compared to methanolic extracts at 200 μg/mL concentration.

#### Superoxide radical scavenging (SARS) assay

All the test extracts exhibited effective O_2_^−*^ scavenging activity in a concentration-dependent manner (10–80 μg/mL), as shown in Figure 9D. The highest activity (Scavenging effect of 72.91 ± 0.76 was shown by methanolic extracts of *D. roylei* at a concentration of 80 μg/mL, followed by ethyl acetate, ethanolic, and petroleum ether extracts with a scavenging effect of 68.125% ± 0.45%, 65.84% ± 0.41% and 62.27% ± 0.5% respectively, which is least as compared to methanolic extract and standard Rutin. Standard Rutin showed the highest scavenging potential of 77.46% ± 0.7% showed the highest potential at 80 μg/mL concentration as compared to all four extracts of the plant.

### *In vitro* anticancer activity

#### MTT results

**FIGURE 10:**
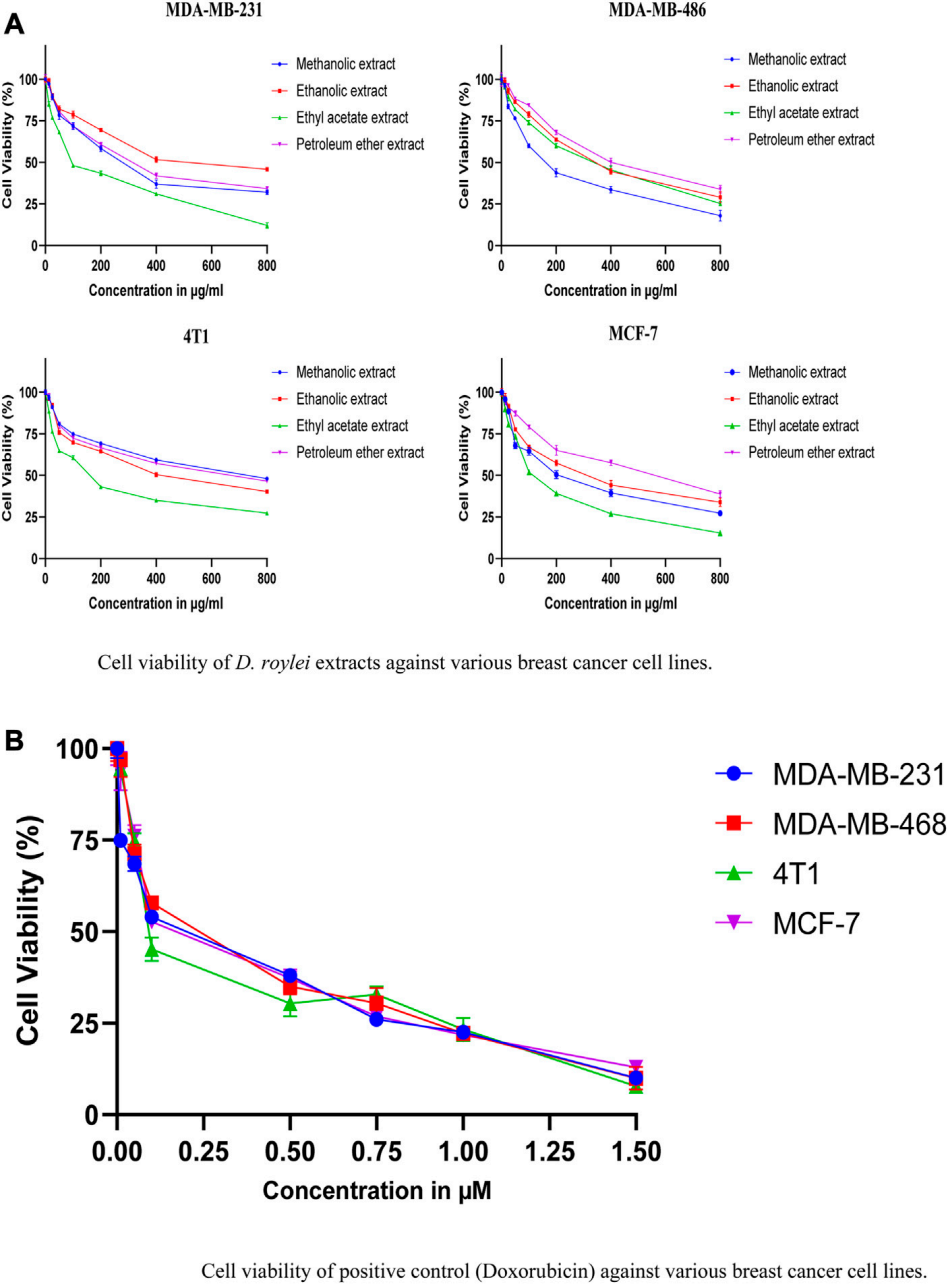
**(A)**. Cell viability of *D. roylei* extracts against various breast cancer cell lines. **(B)** Cell viability of positive control (Doxorubicin) against various breast cancer cell lines.

Figures 10A,B shows the cell viability (%) of various breast cancer cell lines; MDA-MB-231, MCF-7, MDA-MB-468, and 4T1 when treated with different concentrations of methanolic, ethanolic, ethyl acetate, and petroleum ether extracts of *D. roylei* and doxorubicin drug. The IC50 values of four different extracts and positive control against various breast cancer cell lines are shown in Table 7. The ethyl acetate extract of the plant showed a maximum cytotoxic effect on MDA-MB-231 with an IC_50_ value of 116.7 μg/mL, followed by methanolic, petroleum ether, and ethanolic extract with IC_50_ values of 274.1, 317.1, and 549.3 μg/mL respectively. The Methanolic extract of the plant showed maximum reduction in the growth of MD-MB-468 breast cancer cell line with the lowest IC_50_ value of 166.3 μg/mL, followed by ethyl acetate, ethanolic, and petroleum ether extract with IC_50_ values of 296.1, 335.5 and 415.5 μg/mL respectively. The ethyl acetate extract showed maximum anti-proliferative potential against the 4T1 cell line with an IC_50_ value of 157.9 μg/mL. The ethanolic, petroleum ether and methanolic extracts have shown less anti-proliferative potential against 4T1with IC_50_ values of 420.7, 598.9, and 689.4 μg/mL, respectively. The ethyl acetate extract is highly specific to MCF-7 cell lines with an IC_50_ value of 125.5 μg/mL, followed by methanolic, ethanolic, and petroleum ether extracts with IC_50_ values of 213, 299.3, and 498.6 μg/mL, respectively. The IC_50_ values of standard doxorubicin are shown in Table 4. Hence ethyl acetate extract of *D. roylei* showed the highest anticancer activity against the MDA-MB-231, MCF-7 and 4T1 cell lines compared to the other three extracts.

**TABLE 7:** IC_50_ values in μg/mL of various extracts of *D. roylei* against different breast cancer cell lines.

Extracts	MDA-MB-231	MDA-MB-268	4T1	MCF-7
Methanolic extract	274.1	166.3	689.4	213.4
Ethanolic extract	549.3	335.5	420.7	299.3
Ethyl acetate extract	116.5	296.1	157.9	125.5
Petroleum ether extract	317.1	415.5	598.9	498.6
Doxorubicin standard	0.1311	0.1875	0.1433	0.1822

**FIGURE 11:**
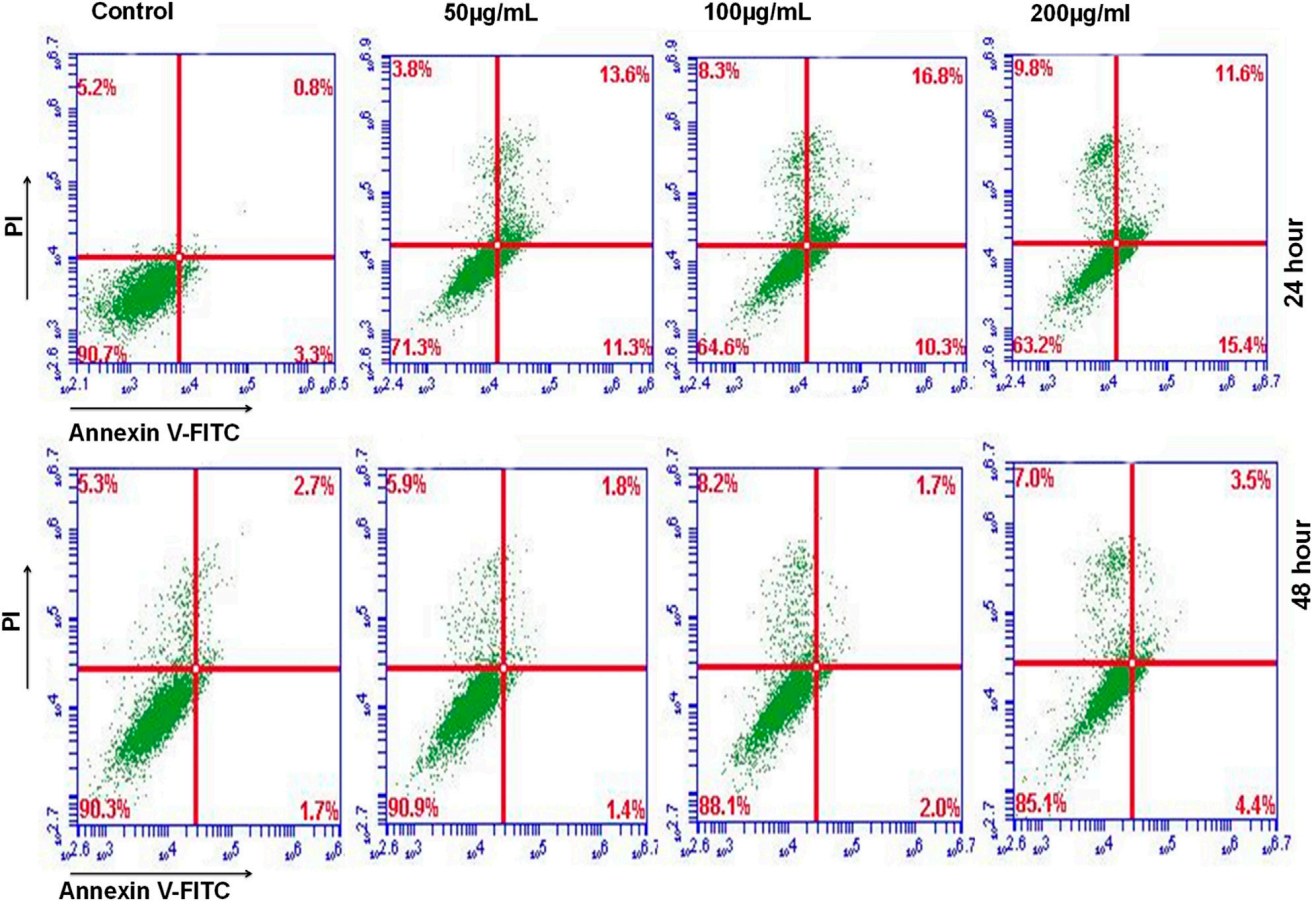
Annexin V & P1 staining showed increased apoptotic cells in plates treated with the ethyl acetate extract of *D. roylei* after 24 h or 48 h periods.

Furthermore, we utilized Annexin V and PI staining to assess the apoptosis induction potential of ethyl acetate extract of *D. roylei*. MDA-MB-231 cells were treated for 24 and 48 h, followed by staining with Annexin V and PI. Flow cytometry analysis revealed that plant extract induction tumour cell death via induction of apoptosis and apoptosis enhanced significantly upon higher concentration are shown in Figure 11.

## Discussion

In the current study, many secondary metabolites were identified using HR-LC/MS of *D. roylei,* out of which few phytocompounds were selected to propose a possible mechanism against breast cancer treatment using network pharmacology, molecular docking, molecular dynamic simulation and *in vitro* studies. The network pharmacology analysis suggested that the therapeutic efficacy of the *D. roylei* phytoconstituents against breast cancer was mainly associated with 20 signalling pathways, 30 potential target genes, and 10 bioactives. Through the network pharmacology, we identified the most significant protein (Akt1) associated with the occurrence and development of cancer and a bioactive 8-hydroxycoumarin (8-hydroxychromen-2-one) from the *D. roylei.* We identified a hub signaling pathway (PI3K-Akt signaling pathway, indicating the lowest rich factor among 20 signaling pathways. Akt1 kinase is a protein made according to instructions from the Akt1 gene (Lv et al., 2020). This protein is present in many different cell types throughout the body and plays a crucial role in numerous signaling pathways. For instance, Akt1 kinase is vital in controlling cell survival, apoptosis, proliferation, and differentiation (Jafari et al., 2019). Recent research has demonstrated that the PI3K/Akt signalling pathways, which play a role in the above-mentioned processes, are frequently disrupted in various human malignancies (Xu et al., 2019). This pathway is crucial for tumor growth and potential responsiveness to cancer treatments.

Many new targeted agents have been created, especially to target P13K/Akt-related targets. Therefore, having a better understanding of the PI3K/Akt signaling pathway may help to enhance the oncologist’s accuracy of prediction as to response to treatment.

Based on the degree value of compounds in the network, we obtained the 8-hydroxycoumarin compound as the most active ingredient of *D. roylei*. Previous studies have revealed that 7-and 4-hydroxycoumarin and its derivatives have numerous therapeutic benefits (Gaber et al., 2021). These are employed as drug intermediates and as antitumor drugs, anti-inflammatory, anti-HIV, antimicrobial, anti-coagulant, antioxidant, and anti-viral agents (Gaber et al., 2021). However, the degree of other compounds was also high, indicating that quality markers may affect the entire biological network system instead of only one target gene.

Furthermore, the KEGG pathway enrichment analysis of 30 targets suggested that 20 top signaling pathways were involved in breast cancer occurrence and development. This indicated that the effect of phytocompounds acts on multiple pathways for treating breast cancer and complex interactions among these pathways. Based on the frequency of each gene in the compound-gene network, Akt1 showed the highest frequency of protein interaction, followed by SRC, MAPK3, EGFR, IL-6, HSP90AA1, ESR-1, and other target genes.

Furthermore, an *in silico* docking analysis of the nine most prevalent compounds was carried out against the 30 potential targets using AutoDock version 4.2.6 software (Morris et al., 2008). All tested compounds showed promising results against Akt1 protein based on docking scores. Among the all, 8-hydroxycoumarin bioactive showed the highest energy score with Akt1. 8-hydroxycoumarin fits comfortably into the binding sites on Akt1 protein and interacts favourably with critical amino acid residues (Figure 7). During the interaction of the ligand 8-hydroxycoumarin, Trp80 is involved in pi-pi stacking, and Lys268 and Val270 residues are involved in pi-alkyl interaction at the binding cavity of the protein. Besides hydrogen bonding, Leu264 is involved in pi-sigma interaction, and the rest residues are van der Waal’s interactions by amino acid residues formed weak non-bonded interaction with the ligand (Figure 7A and right panel). All the binding energy scores are determined from the best cluster (95 percent) that falls within the lowest RMSD 0.25Å. Therefore, it can be inferred from the molecular docking studies that 8-hydroxycoumarin has a high affinity for the protein Akt1.

Also, the stability of the representative Akt1 and 8-hydroxycoumarin complex was further explored using molecular dynamics simulations. The RMSD plots show that the MD results showed stable patterns throughout the entire simulation run (Figures 8A,B). The RMSF graphs show that Akt1 has high flexibility to accommodate the ligand at the binding pocket. The Rg plots demonstrate that the protein stayed compact throughout the simulation. According to the average results observed, the protein backbone was compact. To understand how the residues behaved throughout the simulation run, the fluctuations of the residues were analyzed. After ligand binding, the target’s surface area that was accessible to solvent decreased.

Additionally, our research aimed to find the antioxidant and anticancer potential of *D. roylei* active extracts by *in-vitro* approach. In this investigation, DPPH radical scavenging, Hydroxyl scavenging effect, reducing power, and superoxide radical anion scavenging have shown the antioxidant potential of *D. roylei* extracts and were observed to be significant when compared to positive controls such as Ascorbic acid, BHT, Rutin, and BHT respectively. These observations are in accordance with the previous studies on the antioxidant potential of *Delphinium malabaricum* extracts that the DPPH radical scavenging assay has investigated and the Ferric reducing antioxidant power (FRAP) assay (Lotfaliani et al., 2021)

Further, the petroleum ether, ethyl acetate, methanol, and ethanolic extracts were subjected to cytotoxicity assay against various breast cancer cell lines. The results shown in Figure 10 demonstrated that ethyl acetate extract of *D. roylei* showed the highest anticancer activity against the 4T1, MCF-7, MD-MB-468, and MDA-MB-231 breast cell lines as compared to the other three extracts. As a result, the phytoconstituents found in plant extracts might have a greater propensity to suppress the proliferation of cancer cells. *D. roylei* extracts had relatively lower IC_50_ values against breast cancer cell lines, which may be due to phytocomponents with stronger binding affinities or altering proteins or pathways implicated in tumor development. It can be observed that the ethyl acetate extract had the highest content of the compounds verifying the above-given conclusions about these compounds being the key constituents responsible for the cytotoxic activity of the studied plant. Previous studies by (Yin et al., 2020) revealed that Siwanine E, Uraphine, Delpheline, Delcorinine, Nordhagenine A, Delbrunine, and Delbrunine from *D. honanense* and *D. chrysotrichum* exhibited anticancer potential against MCF-7 and cells with IC_50_ values of 9.62–35.32 μM. Flow cytometry analysis revealed that plant extract induction tumor cell death via induction of apoptosis and apoptosis enhanced significantly upon higher concentration are shown in Figure 11.

## Conclusion

In the present study, we explored the potential mechanisms of phytocompounds present in *D. roylei* in suppressing breast cancer by network pharmacology-based analysis in combination with chemical profiling, molecular docking, MD simulation, and *in vitro* studies. HR-LC/MS identified some important phytoconstituents followed by network pharmacology analysis which revealed that 8-hydroxycoumarin, Delsoline, Royleinine, Delsemine B, Herniarin, Aloesin, Talatisamine, Narwedine, Scoparone, and Piperine were the main constituents related to breast cancer targets while Akt1, SRC, EGFR, IL-6, Hsp-90AA1, and ESR-1 were the main breast cancer-related molecular targets. 20 cancer-related pathways were identified where neuroactive ligand-receptor interaction was the most enriched with the highest number of observed genes and lowest false discovery rate, followed by non-small-cell lung cancer. Molecular docking studies showed that 8-hydroxycoumarin possessed the highest binding energies towards all the target proteins, followed by other compounds against studied targets. Furthermore, *in vitro* studies showed that ethyl acetate extract possess the highest anticancer activity and methanolic extract showed significant antioxidant activity compared to other extracts in the studied plant. The study provided a comprehensive understanding of the suggested mechanism of action of *Delphinium roylei* that may have potential use in breast cancer treatment.

## Data Availability

The original contributions presented in the study are included in the article/Supplementary Material, further inquiries can be directed to the corresponding author.

## Abbreviations

AKT1; RAC-alpha serine/threonine-protein kinase, HR-LC/MS; High Resolution Liquid Chromatography Mass Spectrometry, STRING; Search Tool for the Retrieval of Interacting Genes/Proteins, CDKs; Cyclin Dependent Kinases, RCSB; Research Collaboratory for Structural Bioinformatics, PDB; Protein Data Bank, LGA; Lamarckian Genetic Algorithm, MD; Molecular Dynamic Simulation, RMSD; Root Mean Square Deviation, RMSF; Root Mean Square Fluctuation, OPLS; Optimized Potentials for Liquid Simulations, RG; Radius of Gyration, SASA; Solvent Accessible Surface Area, DPPH; 2,2-diphenyl-1-picrylhydrazyl, MTT; (3-(4,5-Dimethylthiazol-2-yl)-2,5-Diphenyltetrazolium Bromide), GO; Gene ontology, KEGG; (Kyoto Encyclopaedia of Genes and Genomes).
